# Validity and reliability of the Eating Disorder Examination-Questionnaire-7 Portuguese version in the perinatal period

**DOI:** 10.1192/j.eurpsy.2024.263

**Published:** 2024-08-27

**Authors:** A. T. Pereira, R. Lima, D. Pereira, J. M. Pinto, B. Barbosa, A. I. Araújo, C. Marques, A. Macedo, C. Pinto Gouveia

**Affiliations:** ^1^Institute of Psychological Medicine; ^2^Faculty of Medicine, University of Coimbra, Coimbra, Portugal

## Abstract

**Introduction:**

The EDE-Q-7 Portuguese version presented good reliability and validity in Portuguese women fro the general population (Pereira et al. 2022).

**Objectives:**

The aim of our study was to analyse the psychometric properties of the EDE-Q-7 in a sample of Portuguese women during the perinatal period.

**Methods:**

Participants were 346 women with a mean age of 31.68 of years old (± 4.061; range: 18-42). 160 were pregnant (second or third trimester) and 186 were in the post-partum (mean baby´s age=4.37 months (± 2.87; range: 1-12). They answered an online survey including the Portuguese version of the EDE-Q-7 and of the Screen for Disordered Eating/SDE.

**Results:**

Confirmatory factor analysis (CFA) presented adequate fit, in pregnancy (χ2/df=; RMSEA=, p<.001; CFI=; TLI=; GFI=), postpartum (χ2/df=; RMSEA=, p<.001; CFI=; TLI=; GFI=) and considering both – perinatal period (χ2/df=2.7998; RMSEA=.0722, p<.001; CFI=.9709; TLI=.9444; GFI=.9761). The Cronbach’s alpha coefficients were >0.90 for the total and approximately .70 for the three factors - Dietary restraint, Shape/weight overvaluation and Body dissatisfaction. All the items contributed to the internal consistency and presented high internal consistency. Pearson correlations between factors and total scores were significant, positive and high, as well as between the EDE-Q-7 measures and SDE (>.60 with the total; >.40 with the factors), in pregnancy, postpartum and considering both periods.

**Conclusions:**

Presented sound psychometric properties across the perinatal period, the EDE-Q-7 and can be very useful to evaluate the presence and severity of eating disorders symptoms in women in pregnancy and post-partum.

**Disclosure of Interest:**

None Declared

